# Supplementary material for: Non-linear association of cardiometabolic index with gallstone disease in US adults: A cross-sectional study
Source: PLoS One. 2025 Aug 1;20(8):e0328415. doi: 10.1371/journal.pone.0328415 (PMC12316293; doi:10.1371/journal.pone.0328415)
Supplement: S1 Table — (DOCX) [file pone.0328415.s001.docx]

**S1 Table** Association between CMI and gallstone disease (GSD) in NHANES 2017-2020, weighted.

| **Characteristic** | **Model 1** |  |  | **Model 2** |  |  | **Model 3** |  |
| --- | --- | --- | --- | --- | --- | --- | --- | --- |
|  | **OR (95%CI)** | ***P* value** |  | **OR (95%CI)** | ***P* value** |  | **OR (95%CI)** | ***P* value** |
| **CMI** | 1.13 (1.01, 1.27) | **0.041** |  | 1.22 (1.05, 1.41) | **0.012** |  | 1.16 (1.04, 1.30) | **0.015** |
| **Quartile of CMI** |  |  |  |  |  |  |  |  |
| Q1 | Reference |  |  | Reference |  |  | Reference |  |
| Q2 | 2.02 (1.05, 3.88) | **0.036** |  | 1.96 (0.98, 3.90) | 0.056 |  | 1.81(0.78, 4.19) | 0.129 |
| Q3 | 2.16 (0.92, 5.06) | 0.074 |  | 2.09 (0.89, 4.94) | 0.087 |  | 1.87 (0.66, 5.29) | 0.181 |
| Q4 | 2.65 (1.68, 4.19) | **< 0.001** |  | 3.12 (1.92, 5.05) | **< 0.001** |  | 2.56 (1.42, 4.60) | **0.009** |
| ***P* for trend** |  | **< 0.001** |  |  | **< 0.001** |  |  | **0.008** |

Model 1: unadjusted. Model 2: age, sex and race were adjusted. Model 3: age, sex, race, education level, PIR, smoking status, drinking status, physical activity, total cholesterol, hypertension, diabetes, cancer, and coronary heart disease were adjusted. CMI: cardiometabolic index; OR: odds ratio; 95% CI: 95% confidence interval.
