# Supplementary material for: Non-linear association of cardiometabolic index with gallstone disease in US adults: A cross-sectional study
Source: PLoS One. 2025 Aug 1;20(8):e0328415. doi: 10.1371/journal.pone.0328415 (PMC12316293; doi:10.1371/journal.pone.0328415)
Supplement: S2 Table — (DOCX) [file pone.0328415.s002.docx]

**S2 Table** Subgroup analysis between CMI and gallstone disease (GSD).

| **Subgroups** | **OR (95% CI)** | ***P*-value** | ***P* for interaction** |
| --- | --- | --- | --- |
| **Age (year)** |  |  | 0.132 |
| < 50 | 1.12 (0.97, 1.28) | 0.105 |  |
| ≥ 50 | 1.22 (1.05, 1.42) | 0.017 |  |
| **Gender** |  |  | **0.015** |
| Male | 1.09 (0.88, 1.34) | 0.385 |  |
| Female | 1.57 (1.09, 2.25) | 0.021 |  |
| **Education level** |  |  | 0.931 |
| Less than high school | 1.18 (0.93, 1.51) | 0.158 |  |
| High school or GED | 1.21 (0.70, 2.10) | 0.456 |  |
| Above high school | 1.14 (0.99, 1.31) | 0.059 |  |
| **Alcohol** |  |  | 0.788 |
| Yes | 1.11 (0.77, 1.60) | 0.525 |  |
| No | 1.16 (1.01, 1.33) | 0.039 |  |
| **Smoked** |  |  | 0.494 |
| Yes | 1.52 (1.01, 2.27) | 0.045 |  |
| No | 1.10 (0.96, 1.26) | 0.133 |  |
| **Hypertension** | 1.22 (1.05, 1.42) |  | 0.138 |
| Yes | 1.27 (1.08, 1.48) | 0.008 |  |
| No | 1.06 (0.91, 1.24) | 0.391 |  |
| **Diabetes** |  |  | 0.599 |
| Yes | 1.17 (0.97,1.42) | 0.086 |  |
| No | 1.13 (0.98,1.31) | 0.079 |  |
| **Cancer** |  |  | 0.097 |
| Yes | 1.69 (1.14, 2.50) | 0.015 |  |
| No | 1.13 (1.01, 1.26) | 0.037 |  |
| **Coronary heart disease** |  |  | 0.532 |
| Yes | 0.77 (0.21, 2.80) | 0.627 |  |
| No | 1.16 (1.03,1.31) | 0.021 |  |

All covariates (as in Model 3) were adjusted except the stratification variable itself. Abbreviations: CMI: cardiometabolic index; OR, odds ratio; 95% CI; 95% confidence interval.
