# Supplementary material for: Non-linear association of cardiometabolic index with gallstone disease in US adults: A cross-sectional study
Source: PLoS One. 2025 Aug 1;20(8):e0328415. doi: 10.1371/journal.pone.0328415 (PMC12316293; doi:10.1371/journal.pone.0328415)
Supplement: Attachment — Submitted filename: PONE-review.docx [file pone.0328415.s003.docx]

This manuscript investigates the relationship between the Cardiometabolic Index (CMI) and gallstone disease (GSD) in U.S. adults. Using data from the National Health and Nutrition Examination Survey (NHANES) between 2017 and 2020, the study assesses whether CMI (a new composite indicator of obesity and lipid levels) associates non-linearly with GSD risk. The authors reveal that higher CMI correlates with an increased risk of GSD and propose that CMI might serve as a screening tool for GSD, with potential implications for clinical management.

In general, the manuscript is well-written and tables are clear. The authors use multiple ways (logistic regression, restricted cubic spline models, and subgroup analysis) to prove the positive correlation between CMI and GSD. The topic is beneficial to the clinical engagement and clearly suitable for publication in this journal. However, following issues need to be addressed before considering for publication.

1. This manuscript states they use logistics regression and restricted cubic spline models but haven’t provided any formulas or parameters deployed in this study. Need to address the rationale of choosing these two models for evaluating CMI – GSD correlation.

2. Lipid balance is crucial for gallstone disease, why choose CMI as indicator? Not [TG (mmol/L)/HDL-C (mmol/L)]? How does CMI compares to [TG (mmol/L)/HDL-C (mmol/L)] alone? And how does CMI compares to WHtR alone?

2. Lack visuals for logistics regression results and subgroup analysis.

3. Processed data (2825 out of 15,560) is not provided.

4. Although adjustments for multiple covariates were made, some potential confounders like diet habits, physical activity, and genetic predisposition are not accounted for. Discussing their potential impact on the results would improve the study's robustness.

5. Expand bias discussion (lack of other important factors as above). Address limitations of self-reported data -- could introduce recall bias. Highlight this as a limitation and consider suggesting longitudinal studies for validation.

6. Provide more insights on the subgroup analysis in the results, particularly regarding gender differences. Emphasize the clinical implications of gender-based variations in CMI's impact on GSD.

7. While the study indicates that CMI could be a screening tool, it may be helpful to discuss how CMI could complement existing GSD screening practices. Elaborate on how clinicians might integrate CMI in routine evaluations.

8. table 1: need to clearly indicate readers they are “logistic regression” models. The 4^th^ “Q3” should be Q4.

9. figure 2 is too rough. It shows “figure 1” at the top left corner. Need more comprehensive captions explaining all elements including in the plot like the shade and cross-point. Enlarge x-axis and y-axis font size.
