# Supplementary material for: Non-linear association of cardiometabolic index with gallstone disease in US adults: A cross-sectional study
Source: PLoS One. 2025 Aug 1;20(8):e0328415. doi: 10.1371/journal.pone.0328415 (PMC12316293; doi:10.1371/journal.pone.0328415)
Supplement: Attachment — Submitted filename: Comments to Author.docx [file pone.0328415.s004.docx]

**Non-linear association of cardiometabolic index with gallstone disease in US adults: a cross-sectional study**

**Comments to Author:**

The present study demonstrates a nonlinear relationship between higher Cardiometabolic Index (CMI) and an increased risk of gallstone disease (GSD). However, there are several concerns regarding the methods used in the study that merit further consideration

1. What influence does the use of NHANES data, which is cross-sectional in nature, have on how we interpret the relationship between CMI and gallstone disease (GSD)?

2. Would incorporating other biomarkers or cardiometabolic indicators (for example, HOMA-IR or LDL cholesterol levels) into the CMI calculation alter its relationship with gallstone disease?

3. How does the use of waist-to-height ratio (WHtR) as a component of CMI compare to other measures of central obesity, like waist circumference or waist-to-hip ratio, in terms of both benefits and limitations?

4. What is the reliability of using self-reported data from the NHANES questionnaire to diagnose gallstones, and what biases or limitations might arise from relying on self-reported health conditions in epidemiological studies?

5. What are the primary limitations of using a cross-sectional study design to infer causal relationships between CMI and GSD, and how could longitudinal or experimental research designs enhance the validity of these findings?

6. While the study demonstrates a significant link between CMI and GSD, what potential biases (such as selection bias, information bias from self-reported diagnoses, or measurement error) should be considered, and what measures could be implemented in future studies to reduce their impact?

7. The authors propose that CMI could serve as an effective screening tool for identifying individuals at high risk for GSD. How practical is it to implement CMI for this purpose in clinical practice, particularly considering its connection with other metabolic disorders like obesity and diabetes? What limitations might arise, such as issues with cost, accessibility, or applicability across diverse patient populations?
