# Supplementary material for: Non-linear association of cardiometabolic index with gallstone disease in US adults: A cross-sectional study
Source: PLoS One. 2025 Aug 1;20(8):e0328415. doi: 10.1371/journal.pone.0328415 (PMC12316293; doi:10.1371/journal.pone.0328415)
Supplement: Attachment — Submitted filename: Response.docx [file pone.0328415.s006.docx]

Dear Editors and Reviewers:

Thank you for your letter and for the reviewers’ comments concerning our manuscript entitled “**Non-linear association of cardiometabolic index with gallstone disease in US adults: a cross-sectional study**” (ID: 24-47105). Those comments are all valuable and very helpful for revising and improving our paper, as well as the important guiding significance to our research. We have studied the comments carefully and have made a correction. Revised portion are marked in red in the tracked version of manuscript. The final version of our manuscript has been submitted. The main corrections in the paper and the responses to the reviewer’s comments are as flowing:

Responds to the reviewer’s comments:

Reviewer #1:

1. This manuscript states they use logistics regression and restricted cubic spline models but haven’t provided any formulas or parameters deployed in this study. Need to address the rationale of choosing these two models for evaluating CMI – GSD correlation.

Response: We gratefully appreciate your valuable suggestion.We added the rationale of logistics regression and restricted cubic spline models to evaluate the correlation of CMI-GSD in the statistical analysis section of the manuscript.

2. Lipid balance is crucial for gallstone disease, why choose CMI as indicator? Not [TG (mmol/L)/HDL-C (mmol/L)]? How does CMI compares to [TG (mmol/L)/HDL-C (mmol/L)] alone? And how does CMI compares to WHtR alone?

Response: This is a very good suggestion. After synthesizing previous research findings, we posit that compared to individual lipid indices or waist-to-height ratio (WHtR), the cardiometabolic index (CMI) may provide a more comprehensive reflection of human metabolic status. It is noteworthy that we will conduct comparative analyses of these parameters in subsequent multicenter randomized controlled clinical trials to more directly demonstrate the advantages of the CMI index.

3. Lack visuals for logistics regression results and subgroup analysis.

Response: Thanks for your reminder. We have put the forest diagram into Figures 2 and 3.

4. Processed data (2825 out of 15,560) is not provided.

Response: We have uploaded the filtered data to https://figshare.com, DOI: 10.6084/m9.figshare.28323725.

5. Although adjustments for multiple covariates were made, some potential confounders like diet habits, physical activity, and genetic predisposition are not accounted for. Discussing their potential impact on the results would improve the study's robustness.

Response: We think this is an excellent suggestion.We have included physical activity in the covariate and adjusted it to improve the robustness of the results. However, due to the limitations of the NHANES database, diet habits and genetic predisposition are not currently available in the database.

6. Expand bias discussion (lack of other important factors as above). Address limitations of self-reported data -- could introduce recall bias. Highlight this as a limitation and consider suggesting longitudinal studies for validation.

Response: Thank you for the good advice. We have supplemented and highlighted the limitations of this study in the last paragraph of the discussion.

7. Provide more insights on the subgroup analysis in the results, particularly regarding gender differences. Emphasize the clinical implications of gender-based variations in CMI's impact on GSD.

Response: Thank you for your comments. We have added on lines 3 to 7 of page 10.

8. While the study indicates that CMI could be a screening tool, it may be helpful to discuss how CMI could complement existing GSD screening practices. Elaborate on how clinicians might integrate CMI in routine evaluations.

Response: We think this is an excellent suggestion. We have already added lines 250 to 255 in this manuscript.

9. table 1: need to clearly indicate readers they are “logistic regression” models. The 4^th^ “Q3” should be Q4.

Response: Thank you for the good advice. We have supplemented the title in Table 1 and corrected "Q3" to "Q4" in Table 2.

10. figure 2 is too rough. It shows “figure 1” at the top left corner. Need more comprehensive captions explaining all elements including in the plot like the shade and cross-point. Enlarge x-axis and y-axis font size.

Response: Thank the reviewer for reading our paper carefully and giving the above positive comments. We have reworked Figure 4 and supplemented it in the first paragraph on page 8.

Reviewer #2

1. What influence does the use of NHANES data, which is cross-sectional in nature, have on how we interpret the relationship between CMI and gallstone disease (GSD)?

Response: This is a very good suggestion. We must admit that there are some inherent limitations to this study. Because this study is a cross-sectional study based on NHANES data, a causal relationship between CMI and GSD cannot be determined. However, in the follow-up study, our team will conduct a multicenter randomized controlled clinical trial to verify the relationship between the two.

2. Would incorporating other biomarkers or cardiometabolic indicators (for example, HOMA-IR or LDL cholesterol levels) into the CMI calculation alter its relationship with gallstone disease?

Response: We think this is an excellent suggestion. Some previous studies have found that HOMA-IR and LDL cholesterol levels are closely related to obesity and lipid metabolism, but there are no appropriate calculation methods and studies to incorporate these indicators into CMI calculation. In previous studies, HOMA-IR and LDL cholesterol levels have been found to be significantly associated with the risk of gallstones. Therefore, combined with our study results, we concurred that the inclusion of HOMA-IR and LDL cholesterol levels in CMI calculation would not change their relationship with gallstones, but this needs to be verified by multi-center randomized controlled clinical trials.

3. How does the use of waist-to-height ratio (WHtR) as a component of CMI compare to other measures of central obesity, like waist circumference or waist-to-hip ratio, in terms of both benefits and limitations?

Response: The waist-to-height ratio (WHtR) eliminates potential biases in the assessment of central obesity caused by height by standardizing waist circumference relative to height. Compared to waist circumference (WC) or waist-to-hip ratio (WHR) alone, WHtR provides a more equitable measure of obesity for populations with significant height variations, such as individuals from different ethnic groups. Studies have shown that WHtR exhibits higher sensitivity and specificity than WC and WHR in predicting the risks of metabolic syndrome, cardiovascular diseases, and diabetes. However, it is important to acknowledge the limitations of WHtR. First, for individuals with extremely tall or short stature, WHtR may underestimate or overestimate obesity risk, necessitating the use of additional indicators (e.g., BMI) for a comprehensive assessment. Second, WC and WHR remain the core metrics recommended by most clinical guidelines, and the clinical application of WHtR has yet to be widely adopted.

4. What is the reliability of using self-reported data from the NHANES questionnaire to diagnose gallstones, and what biases or limitations might arise from relying on self-reported health conditions in epidemiological studies?

Response: Thank you for your comments. In this study, the use of self-reported data from the NHANES questionnaire as the diagnostic criterion for gallstones was based on prior research. However, it is important to acknowledge that reliance on self-reported health conditions in epidemiological studies may introduce biases such as recall bias (e.g., participants may forget or misinterpret diagnostic details) and potential underdiagnosis or misdiagnosis.

5. What are the primary limitations of using a cross-sectional study design to infer causal relationships between CMI and GSD, and how could longitudinal or experimental research designs enhance the validity of these findings?

Response: Thank you for the good advice. First, in a cross-sectional study, both the exposure (CMI) and the outcome (gallstones) are measured simultaneously, making it impossible to determine whether the exposure precedes the outcome. Second, although we controlled for known confounding factors through multivariate adjustments, unmeasured confounders (such as genetic predisposition and dietary patterns) may still influence the results. Lastly, cross-sectional studies only provide a snapshot at a single point in time and cannot reflect the dynamic relationship between cardiometabolic indices and gallstones. In a longitudinal study design, long-term follow-up can help determine whether the exposure precedes the outcome. Additionally, repeated measurements can reveal the dynamic association between metabolic indicators and the risk of gallstones. In experimental study designs, randomized controlled trials, through random allocation, can minimize confounding bias and directly assess the impact of interventions (such as improving metabolic indicators) on gallstone risk. Furthermore, Mendelian randomization can also be used to enhance the reliability of causal inference.

6. While the study demonstrates a significant link between CMI and GSD, what potential biases (such as selection bias, information bias from self-reported diagnoses, or measurement error) should be considered, and what measures could be implemented in future studies to reduce their impact?

Response: We think this is an excellent suggestion. First, this study should consider selection bias: the general community population may not be representative of hospital-based cohorts. Second, information bias: self-reported diagnoses of gallstones may introduce recall bias, particularly since asymptomatic cases are likely to be underreported. Finally, measurement error: errors may arise from the measurement of waist-to-height ratio. In future research, we can track the temporal changes in the development of CMI and gallstones to clarify the temporal sequence and dynamic relationships. Additionally, combining evidence from observational studies, randomized controlled trials, and Mendelian randomization can enhance the robustness of causal inference.

7. The authors propose that CMI could serve as an effective screening tool for identifying individuals at high risk for GSD. How practical is it to implement CMI for this purpose in clinical practice, particularly considering its connection with other metabolic disorders like obesity and diabetes? What limitations might arise, such as issues with cost, accessibility, or applicability across diverse patient populations?

Response: We sincerely thank the editor and all reviewers for their valuable feedback that we have used to improve the quality of our manuscript. We have supplemented the clinical application of CMI in the second paragraph of page 9.

Reviewer #3

1. This research is aligned with other research on cardiometabolic disease and liver disease such as GSD metabolic-associated fatty liver disease (MAFLD), applying different indices and statistical methods to test the association. The biological mechanism and underlying assumptions regarding the associations have been clearly defined previously, and possible co-variates/confounders have been identified, but the questions regarding which CM index is the most sensitive and specific clinical indicator for possible imminent liver disease in different populations residing in different environments and whether a valid universal threshold may be derived from the analyses if the association between the outcome and the index is not linear are not yet been answered. Although this study applied weighting in their analysis and adjusted for all relevant confounders in the multivariate logistic regression analyses, linearity was not observed. Restricted cubic spline analyses were thus performed to test for a ‘dose-dependent’ relationship, but failed to identify a threshold.

Response: Thank you for your comments.We conducted a threshold effect analysis of the relationship between CMI and GSD and included the results in Table 3.
