# Supplementary material for: Non-linear association of cardiometabolic index with gallstone disease in US adults: A cross-sectional study
Source: PLoS One. 2025 Aug 1;20(8):e0328415. doi: 10.1371/journal.pone.0328415 (PMC12316293; doi:10.1371/journal.pone.0328415)
Supplement: Attachment — Submitted filename: Response_auresp_2.docx [file pone.0328415.s007.docx]

Dear Editors and Reviewers:

Thank you for your letter and for the reviewers’ comments concerning our manuscript entitled “**Non-linear association of cardiometabolic index with gallstone disease in US adults: a cross-sectional study**” (ID: 24-47105). Those comments are all valuable and very helpful for revising and improving our paper, as well as the important guiding significance to our research. We have studied the comments carefully and have made a correction. Revised portions are marked in red in the tracked version of manuscript. The final version of our manuscript has been submitted. The main corrections in the paper and the responses to the reviewer’s comments are as flowing:

Responds to the reviewer’s comments:

Reviewer #1:

1. Lipid balance is crucial for gallstone disease, why choose CMI as indicator? Not [TG (mmol/L)/HDL-C (mmol/L)]? How does CMI compares to [TG (mmol/L)/HDL-C (mmol/L)] alone? And how does CMI compares to WHtR alone?

Response: We sincerely thank the editor and all reviewers for their valuable feedback that we have used to improve the quality of our manuscript. We used ROC analysis to compare CMI with WHtR and TG/HDL and added this analysis to the article.

Reviewer #3

1. Consider making more detailed and stronger deductions from the observed results, e.g., the subgroup analyses that were conducted to test for interactions between CMI and specific covariates. What stood out was the preponderance of males rather than females in the highest quartile (62.16 vs 37.8%) and the increasing trend in male proportions across quartiles with a reversed trend in females. This may be highlighted as a novel finding.

Response: We sincerely appreciate the insightful observation. We've added this novel finding to the results section.

2. There are numerous syntax errors throughout that need attention. The Lipid Accumulation Product (LAP) index is mentioned in a sub-title within the Results section. This is the only occurrence of the term. Please clarify its relevance, because the paragraph reports on the CMI and the formulas for the two indices’ calculation differ.

Response: Thanks for your reminder. That was a writing error, and we've changed it to CMI.

3. The first paragraph in the introduction addresses Gallstone disease in general. The references are rather outdated. Please consider replacing them or adding more recent references (some of which are included in the above list of references that may be of interest to the authors)

Response: We gratefully appreciate your valuable suggestion. We have added some of the literature from the above list of references to the first paragraph of the introduction.

4. In the third paragraph of this section, the CMI is introduced. You may consider referring to other more recently applied indices and state why you chose the CMI. E.g., that it has better predictive potential, as alluded to in the discussion section You refer to some of these indices in the paragraph within the discussion section that starts with ‘The CMI, as an indicator to assess the degree of obesity and lipid levels…’ but referring to them in the introduction section reflects insight within this field. Consider re-writing the Conclusion so that it captures the contribution of this study to the body of scientific knowledge more distinctly.

Response: Thank the reviewer for reading our paper carefully and giving the above positive comments. We have added these recommendations in the third paragraph of the introduction and rewritten the Conclusion.
