# Supplementary material for: Non-linear association of cardiometabolic index with gallstone disease in US adults: A cross-sectional study
Source: PLoS One. 2025 Aug 1;20(8):e0328415. doi: 10.1371/journal.pone.0328415 (PMC12316293; doi:10.1371/journal.pone.0328415)
Supplement: Attachment — Submitted filename: Response_auresp_3.docx [file pone.0328415.s008.docx]

Dear Editors and Reviewers:

Thank you for your letter and for the reviewers’ comments concerning our manuscript entitled “**Non-linear association of cardiometabolic index with gallstone disease in US adults: a cross-sectional study**” (ID: 24-47105). Those comments are all valuable and very helpful for revising and improving our paper, as well as the important guiding significance to our research. We have studied the comments carefully and have made a correction. Revised portions are marked in red in the tracked version of manuscript. The final version of our manuscript has been submitted. The main corrections in the paper and the responses to the reviewer’s comments are as flowing:

Responds to the reviewer’s comments:

Reviewer #3:

1. “In contrast (line 98) to previous BRI and LAP indicators, CMI …” Please precede the acronyms with the full terms.

Response: We sincerely thank the editor and all reviewers for their valuable feedback that we have used to improve the quality of our manuscript. We have added the full terms before the acronym

2. Line 276: Consider replacing ‘literatures’ with ‘reports’.

Response: Thanks for your reminder. We have made corrections

3. In Vancouver style, "P-value" should be written with a capital "P" and without a hyphen, as "P value".

Response: We sincerely appreciate the insightful observation. We change all the "P-value" in the manuscript to "P-value".

General comment:

4. Line 309: Consider preceding the Conclusion with a summative statement regarding the clinical relevance of these findings, such as: "However, the clinical relevance of identifying emergent GSD using the CMI is extended by acknowledging the significant relationship between GSD and CVD, probably due to common causal pathways, as established through meta-analysis and meta-regression."

Response: Thank the reviewer for reading our paper carefully and giving the above positive comments. We have added these recommendations in line 276.
